# Internet addiction: a 21^st ^century epidemic?

**DOI:** 10.1186/1741-7015-8-61

**Published:** 2010-10-18

**Authors:** Dimitri A Christakis

**Affiliations:** 1 Center for Child Health, Behavior, and Development, Seattle Children's Research Institute, Seattle, Washington, USA; 2 Department of Pediatrics, University of Washington, Seattle, Washington, USA

## Abstract

Internet addiction, while not yet officially codified within a psychopathological framework, is growing both in prevalence and within the public consciousness as a potentially problematic condition with many parallels to existing recognized disorders. The rapid and unfettered increase in the number of people accessing a relatively unrestricted internet substantially increases the possibility that those suffering with an underlying psychological comorbidity may be at serious risk of developing an addiction to the internet, lending further credence to this hitherto understudied condition. In this commentary, I outline my recommendations for improved diagnosis, study and prevention of internet addiction.

## Background

Lee Seung Seop achieved his 15 minutes of fame in a most tragic fashion. The 28-year-old boiler repairman suffered a cardiac arrest following a 50-hour internet gaming binge during which he neither ate nor slept. His death prompted an investigation into the problem of internet addiction in Korea, where current estimates are that 4% of children suffer from the disorder [1]. International estimates for children vary widely, with European prevalence reported at between 1 and 9% [2-6], Middle Eastern prevalence at between 1 and 12% [7-9] and prevalence in Asia reported between 2 and 18% [10-17]. However, these estimates must be interpreted with some caution, as varying scales with questionable validity and conflicting reports make true generalizations difficult. Additionally, the field has been hampered by methodological weaknesses of existing research, among which the most salient has been sampling bias. Many early studies relied on voluntary internet surveys without measurable denominators, convenience samples of internet users or chat room sampling [18,19], but even beyond issues of sampling, there is the thorny debate about whether internet addiction exists at all as an entity. It is currently not a formally recognized disorder, although internet addiction is being considered for the forthcoming DSM-V [20].

## Discussion

Regardless of whether internet addiction is codified within a psychopathological framework, it is fair to say that there is, at the least, the potential for a problem. In the absence of formal diagnostic criteria, most researchers currently model problematic internet usage on problematic gambling, extrapolating from one compulsive nonpharmacologically addictive behavior to another. Key components of addiction include preoccupation with the substance or behavior; repeated unsuccessful attempts to reduce it; mood disturbances related to reduction attempts; greater usage than anticipated or desired; jeopardizing employment, relationships or education; or lying about usage. All of these criteria, at least theoretically, can be seen with internet usage. In fact, in most scholarly circles, given the strong theoretical basis to believe that there could be a problem with pathological use of the Internet, the debate is not so much about whether it exists but about just how prevalent it is.

However, it should be noted that there are significant ways in which likening internet addiction to problematic gambling is inadequate. All existing behaviors or substances that have been shown to lead to addiction, such as alcohol, gambling, tobacco or drugs, have structural constraints on their usage either by law or by social etiquette. Consuming alcohol at the office or at school is sure to land one in trouble; conversely, instant messaging or surfing the web is likely not to. Cultural influences both mandate and facilitate that we spend time "online," meaning that teetotalism is not an option. Given our current understanding that there is a genetic predisposition to behavioral addictions, we may be going a long way toward ensuring that the entire susceptible population develops them. Existing research on internet addiction has also highlighted specific subpopulations that are at increased risk, including those with other psychological comorbidities, including attention-deficit/hyperactivity disorder (ADHD), depression and social isolation [21-25]. These risk factors lend further credence to the validity of the disorder, since they have also been associated with other behavioral addictions as well as substance abuse [26-28].

As this generation of youth is leading the transition to a fully wired society [29], they deserve adequate safeguards and protections against the attendant risks of the technological revolution. Here is what is needed, and quickly. First, a definition of internet addiction that is both validated and applicable to children and adolescents alike should be developed. Existing measures designed for adults have not been well validated in adolescents and many of the domains (e.g., interfering with household chores or schoolwork) are of limited applicability to these populations. Second, we need a better scientific understanding of which types of usage pose the greatest risk of addiction. For example, as with television, it is likely that it is not merely the amount of time that is spent online but *how *it is spent that mediates its effects [30]. Virtual reality games in particular, where participants assume other identities or collaborate with team members all over the globe, may pose the greatest risk of addiction, since frequent and continuous online presence is both vital and expected; moreover, going offline can have penalties associated with it. The profit margin of these new, subscription-based games is based entirely on keeping people playing and therefore paying. Purveyors of these products therefore have a perverse incentive to develop addictive games. Third, effective primary prevention strategies need to be developed, tested and implemented. Limits on screen time of all types are important for all children, but in the advent of ubiquitous access these are increasingly difficult to enforce [31]. Providers, parents and teachers require approaches that are proven effective and that allow for necessary and even healthy internet usage. Fourth, a targeted prevention approach, identifying children at greatest risk for addiction, is also important. Children with preexisting psychosocial morbidities may be at greatest risk, and their internet usage should be more explicitly monitored and regulated by guardians and protectors.

## Conclusion

In my opinion, the greatest concern facing this field of research is a general complacency toward internet addiction. In some cases, this complacency is born of ignorance. Too many parents are simply unaware of what their children are doing online and what risks it might pose. In the 20th century, commentators spoke of a digital divide. In those days, it existed along economic lines. That divide has narrowed, or even disappeared, but the 21^st ^century digital divide separates parents from their children. In other cases, the complacency is born of skepticism. In much the same way that tobacco proponents pushed back on early research linking smoking to cancer, these skeptics are quick to point out limitations of existing research as a way of casting doubt on the entire field. There is no question that the field of internet addiction research is in its infancy and that the overall quality of existing data is fair to moderate at best, but that should not distract us or prevent us from taking what is an emerging problem seriously.

## Abbreviations

DSM: Diagnostic and Statistical Manual of Mental Disorders; ADHD: attention-deficit/hyperactivity disorder.

## Competing interests

The authors declare that they have no competing interests.

## Authors' contributions

DAC is entirely responsible for the content of this article.

## Pre-publication history

The pre-publication history for this paper can be accessed here:

http://www.biomedcentral.com/1741-7015/8/61/prepub
